# Diagnostic Work-Up of the Aortic Patient: An Integrated Approach toward the Best Therapeutic Option

**DOI:** 10.3390/jcm10215120

**Published:** 2021-10-31

**Authors:** Michele Pighi, Davide Giovannini, Roberto Scarsini, Nicolo Piazza

**Affiliations:** 1 Division of Cardiology, Department of Medicine, McGill University Health Center, Montreal, QC H4A 3J1, Canada; nicolopiazza@mac.com; 2 Division of Cardiology, Department of Medicine, University of Verona, 37126 Verona, Italy; davide.giovannini21@gmail.com (D.G.); roberto.scarsini@aovr.veneto.it (R.S.)

**Keywords:** TAVI, aortic stenosis, work-up

## Abstract

Aortic stenosis (AS) is the most common valvular heart disease. In the last decade, transcatheter aortic valve implantation (TAVI) has become the standard of care for symptomatic patients at high surgical risk. Recently, indications to TAVI have also been extended to the low surgical risk and intermediate surgical risk populations. Consequently, in this setting, some aspects acquire greater relevance: surgical risk evaluation, clinical assessment, multimodality imaging of the valve, and management of coronary artery disease. Moreover, future issues such as coronary artery re-access and valve-in-valve interventions should be considered in the valve selection process. This review aims to summarize the principal aspects of a multidimensional (multidisciplinary) and comprehensive preprocedural work-up. The Heart Team is at the center of the decision-making process of the management of aortic valve disease and bears responsibility for offering each patient a tailored approach based on an individual evaluation of technical aspects together with the risks and benefits of each modality. Considering the progressive expansion in TAVI indication and technological progress, the role of a work-up and multidisciplinary Heart Team will be even more relevant.

## 1. Introduction

Aortic stenosis (AS) is the most common valvular heart disease in developed countries [1].

Transcatheter aortic valve implantation (TAVI) is a percutaneous technique that, over the last decade, has become the standard of care for patients presenting with symptomatic severe aortic stenosis at high or intermediate surgical risk, and its use is extending quickly to lower risk groups [2]. In high-income health care systems, driven by rapid improvements in both technique and equipment, TAVI procedures have increased more than twenty-fold to become a more frequently performed procedure than surgical aortic valve replacement (SAVR) since 2013 [3].

In this setting, given the results of a randomized controlled trial [4,5] in the low and intermediate low-risk population, we are witnessing the demographic characteristic changes and the reduction in the surgical risk score in the TAVI population. 

Consequently, some new aspects in diagnostic work-up and the aortic valve replacement planning have become crucial. In particular, the evaluation of coronary artery disease and its percutaneous treatment and its timing, related to the TAVI procedure, should also be considered in a future perspective. Given the younger age of TAVI patients, the possibility of undergoing a percutaneous coronary intervention over the years has increased. Therefore, the coronary artery access across the bioprosthetic cage should be a central role point in the valve prosthesis decision-making process.

The patient selection remains crucial in determining whether a patient is likely to benefit from catheter-based intervention instead of surgical intervention or to assess the possible futility of the intervention. Several clinical, anatomical, and procedural aspects need to be considered by the multidisciplinary team (Heart Team) to decide which specific approach is best for treating severe aortic valve disease in each patient [6,7].

The present review aims to summarize the main steps of the work-up process leading to TAVI, and discusses the most challenging aspects the Heart Team will face in the future.

## 2. Initial Clinical Assessment: Surgical Risk, Comorbidities, and Physical Frailty

### 2.1. The Surgical Risk Evaluation

Initially, TAVI was indicated for patients of any age with symptomatic severe calcific aortic stenosis at high or prohibitive surgical risk [8]. High and prohibitive surgical risk is defined as follows: STS-predicted risk of death >8%, moderate or severe frailty, assessed using Katz Activities of Daily Living Score, or one or more organ systems compromised, not to be improved postoperatively [8].

Currently, TAVI is considered as an effective therapeutic option in patients at intermediate surgical risk and is expanding to patients at low surgical risk [4,7,9].

The most commonly used surgical risk scoring systems for assessing patients before TAVI are EuroSCORE I/II, the logistic EuroSCORE, and the Society of Thoracic Surgeons (STS) risk score.

EuroSCORE I was derived from the 1995 regional surgical outcomes of a cohort of nineteen thousand patients who underwent cardiac surgery under cardiopulmonary bypass [10].

EuroSCORE II was based upon an updated worldwide dataset that included one-third of patients who underwent aortic valve replacement [11].

The STS score is derived from a U.S. dataset and presents a dedicated sub-model for six specific surgical procedures including surgical isolated aortic valve replacement [12]. A recently introduced STS/ACC TAVI risk score, based on the STS/ACC TVT Registry, that captures all commercial TAVI procedures performed in the USA, has been developed for predicting in-hospital mortality after TAVI [13].

In a recent study based on a German all-comers registry, when compared with other existing risk scores, the STS/ACC TAVI and STS-PROM showed superiority in predicting 30-day mortality [14].

Risk scores were developed based on datasets from heterogeneous groups of patients who underwent cardiac surgery; therefore, they present some inherited limitations when applied to TAVI patients. Risk scores on their own cannot provide a global and complete evaluation of the surgical risk [15]. Indeed, while some patients may be eligible for SAVR according to the risk score, some characteristics such as porcelain aorta, previous chest radiation therapy, and frailty not included in the scores may lead patients to be referred to TAVI [6] (Table 1).

The recent guidelines, developed by the Task Force for the management of valvular heart disease of the European Society of Cardiology (ESC) and the European Association for CardioThoracic Surgery (EACTS), recommend TAVI in patients older than 75 years or in those who are at high surgical risk or unsuitable for surgery (Class I; level A) [7]. Conversely, surgical aortic valve replacement (SAVR) is recommended in younger patients (<75 years old) and at low risk for surgery (STS-PROM/EuroSCORE II <4%) (Class I; level B) [7]. The choice between surgical and transcatheter intervention in patients younger than 75 years old at intermediate surgical risk must be based upon careful evaluation of clinical, anatomical, and procedural characteristics [7].

Therefore, in this field, the multidisciplinary Heart Team evaluation remains the cornerstone of the decision-making process.

### 2.2. Clinical/Frailty Assessment

An objective assessment of patient frailty still represents a challenging step in the preprocedural work-up. Frailty is a syndrome defined as three or more deficits in geriatric domains including slowness, weakness, unintentional weight loss, exhaustion, and inactivity [16].

The assessment of frailty should not be based on a subjective approach but rather on a combination of different objective evaluations [7] (Table 2).

Disability is defined as one or more dependencies in activities of daily living (ADLs), which include bathing, dressing, toileting, mobilization, continence, and feeding (Katz Index) [17,18].

It is also important to distinguish frailty from comorbidity, which relates to the presence of disease in more than one organ system. The Charlson comorbidity index is a weighted index that considers the number and the seriousness of comorbid disease [19]. Frailty assessment and prediction of poor outcomes are more difficult in the elderly, medically complex, and heterogeneous TAVI population than SAVR patients. 

Frailty is associated with increased 1-year mortality after TAVI and a higher risk of poor outcome [20]. There is still a lack of consensus in the literature on how frailty should be assessed, and many instruments and scales have been proposed. The FRAILTY-AVR study found out that in a cohort of TAVI patients assessed with seven different frailty tools, the prevalence of frailty ranged from 26% to 68% [21]. The following frailty scales were compared: Fried, Fried+, Rockwood Clinical Frailty Scale, Short Physical Performance Battery, Bern, Columbia, and the Essential Frailty Toolset (EFT).

In particular, EFT, a brief four item scale based on lower-extremity weakness, cognitive impairment, anemia, and hypoalbuminemia, outperformed other frailty scales [21] in such a setting.

Frailty assessment adds useful information for the prediction of midterm mortality and also progressive disability following TAVI [21]. Indeed, functional decline and poor patient-centered outcomes at the 1-year follow-up were found in more than 50% of patients deemed frail [21]. The comprehensive geriatric assessment frailty index (CGA-FI) is calculated by the proportion of deficits among 48 health-related items in multiple domains [22]. A comprehensive geriatric assessment was proposed specifically to classify frailty status and predict disability after TAVI. CGA-FI improves prediction over a traditional cardiac surgery-specific assessment [23]. However, such a score alone cannot detect futility; indeed, in medically complex TAVI patients, functional impairment, mainly driven by severe symptomatic aortic stenosis, may recover post-procedure [24]. Conversely, EFT was found to best predict therapeutic futility, with eight out of 10 patients with EFT scores 5/5 experiencing fatal or disabling outcomes at 1-year follow up [21].

In the TAVI work-up, especially for high-risk patients, the futility assessment, defined as a lack of medical efficacy, should be taken into account. Such a strategy is of particular value when the index therapy is unlikely to produce its intended clinical result and requires a multi-faceted evaluation integrating different information [25]. Futility should be considered and accordingly estimated appropriately in the presence of one of the following: an STS score >15; life expectancy <1-year, poor candidate for rehabilitation. 

Non-cardiac conditions related to poor post-TAVI outcomes and futility are severe chronic lung, kidney, and liver diseases [26]. A broad-spectrum evaluation is needed because cognitive dysfunction and malnutrition [27] both predict poor prognosis in patients undergoing TAVI.

Reduced left ventricular ejection fraction, low valve gradients, and reduced stroke volume are related to poor outcomes and futility [25]. Severe pulmonary hypertension and concomitant heart valve disease should be considered and added in the prognostic metric of frailty to avoid such procedures in frail subjects [26].

## 3. Multimodality Imaging Assessment of Aortic Valve Stenosis

### 3.1. Transthoracic Echocardiography 

Medical and interventional management of patients with valvular aortic stenosis depends on accurate diagnosis and staging of the disease process. Echocardiography is the key diagnostic tool^6^, and Doppler echocardiography is the preferred and most available noninvasive technique for assessing the aortic stenosis severity [28] (Table 3). Although echocardiography allows for reliable aortic valve evaluation [28], some patients require further evaluation to accurately diagnose the disease. To achieve a comprehensive assessment, a patient presenting with aortic stenosis should be referred to a highly specialized Heart Valve Center [8].

Key aspects to consider are aortic valve area, flow rate, mean pressure gradient, ventricular function, ventricular wall thickness, degree of valve calcification, and blood pressure.

The latest is of particular importance since the severity of AS should ideally be assessed during normotensive conditions. Conversely, hypertension may underestimate the stenosis severity by imposing a second pressure load on the left ventricle [28]. 

Valve area, calculated with the continuity equation, theoretically represents the ideal measurement for assessing aortic stenosis severity. Nevertheless, such an approach shows some limitations in clinical practice. The Doppler evaluation (transaortic maximum velocity and mean pressure gradient, calculated with the Bernoulli equation) represents the most reliable parameter to characterize the hemodynamic severity [8].

The flow rate evaluation is crucial in the stenosis severity assessment, and meticulous attention is required to avoid underestimating AS severity.

In some cases, patients present with a condition called “low-flow/low-gradient” AS, which is characterized by low transaortic volume flow rate (defined by a stroke volume index (SVi) ≤35 mL/m^2^), either because of left ventricular systolic dysfunction (LVEF <50%), associated with a low ejection fraction, or a small and hypertrophic left ventricle. The latter typically characterizes the elderly, presenting with small ventricular size, marked LV hypertrophy, and hypertension [29].

In contrast, patients presenting with a paradoxical low-flow/low-gradient AS have a normal LVEF with a low transvalvular velocity and pressure gradient (velocity <4 m/s or mean gradient <40 mmHg) at rest. The diagnosis of severe aortic stenosis in this setting remains particularly challenging. The quantification of aortic valve calcification by the computed tomography calcium score is especially useful in this context. Sex-specific Agaston unit thresholds for diagnosis of severe AS are 1300 in women and 2000 in men. These different thresholds reflect the contribution of leaflet fibrosis and calcification to the increased leaflet stiffness in women [8]. The degree of aortic valve calcification is a strong clinical predictor [30].

In patients with severe AS characterized by low transaortic velocity, low-pressure gradient, and impaired LVEF <50%, it is key to differentiate between LV systolic dysfunction due to afterload mismatch and primary myocardial dysfunction associated with only moderate AS [8].

In this scenario, a low dose dobutamine stress echocardiography may help distinguish truly severe aortic stenosis from pseudo-severe aortic stenosis. Severe aortic stenosis is defined by a fixed valve area (<1.0 cm), increasing transvalvular aortic velocity.

In contrast, in patients with pseudo-severe AS stress, echocardiography shows an increase in the valve area as the volume flow rate increases, leading to a modest increase in transaortic velocity or gradient [31,32].

Low dose dobutamine stress echocardiography also adds prognostic information. Indeed, contractile reserve detection, defined as an increase in stroke volume ≥20% during stress, is associated with better clinical outcome [33,34].

Echocardiographic assessment of the aortic valve area (AVA) is based on continuity-equation. It requires three variables, which are aortic stenosis jet velocity, calculated by continuous-wave Doppler (CWD), left ventricle outflow tract (LVOT) diameter, and LVOT velocity recorded by pulsed Doppler. 

Errors in the calculation of AVA could be derived from variability in one of the three variables/measurements. However, to obtain the cross-sectional area of LVOT, the diameter is squared. Indeed, it becomes the principal and greatest potential source of errors in the continuity equation [28]. Moreover, in the continuity equation, the LVOT area is considered to have a circular shape, although it has been recognized that it is more elliptical [35]. 3D echocardiography permits correctly evaluate LVOT area due to a direct planimetry measurement, avoiding underestimation of AVA [36]. Other common sources of errors are the incorrect position of the pulsed-Doppler in the LVOT, the non-alignment of the CWD beam with the flow across the aortic valve.

An alternative to the continuity-equation aortic valve area (AVA) is the visualization of the valve orifice and the direct valve planimetry. Transesophageal echocardiography (TEE) allows direct and accurate valve planimetry measurements, which correlate with invasive data and planimetry by MSCT [37,38].

Although the direct planimetry prevents errors in the continuity-equation derived AVA, effective, rather than anatomic, valvular orifice area is the primary predictor of clinical outcomes [28]. Moreover, due to the contraction of the flow stream in the orifice, the effective orifice area (EOA) is significantly smaller than the anatomic orifice directly measured by the planimetry valve area [39].

When the assessment of aortic stenosis severity presents some inconsistencies, the left ventricle outflow tract–to–aortic velocity ratio may be a useful decisional tool since it is independent of body size and LV outflow diameter. A ratio <0.25 corresponds to a valve area of 25% of normal for that patient, which is consistent with severe AS and is a predictor of symptom onset and adverse outcomes [40].

Cardiac magnetic resonance (CMR) can also be used in cases where echocardiographic results are inconclusive.

Recently, artificial intelligence (AI) has been applied in electrocardiogram (ECG) analysis to precocious identification of moderate or severe AS patients [41]. The application of AI, combining a deep learning-based algorithm and convolutional neural network [42], to diagnose severe aortic stenosis could be a promising tool in the near future.

### 3.2. ECG-Gated Computed Tomography 

During transcatheter aortic valve implantation, as opposed to surgical aortic valve replacement, there are no benefits derived by the direct aortic valve visualization and prosthesis sizing. Consequently, the acquisition of good-quality pre- and periprocedural imaging is a mandatory step to achieve satisfactory results.

In the formative years of TAVI, annular measurements were performed using two-dimensional transthoracic/transesophageal echocardiography or calibrated aortic angiography [43]. Compared to calibrated aortic angiography, transthoracic echocardiography underestimates the annulus dimensions by 1.5 mm ± 2.3 mm [44], while there is no relevant systematic difference between transthoracic echocardiography (TTE) and TEE^46^.

The limitations of two-dimensional measurement techniques are mainly related to the noncircular anatomical configuration of the annulus [45,46,47].

**Figure 1:**
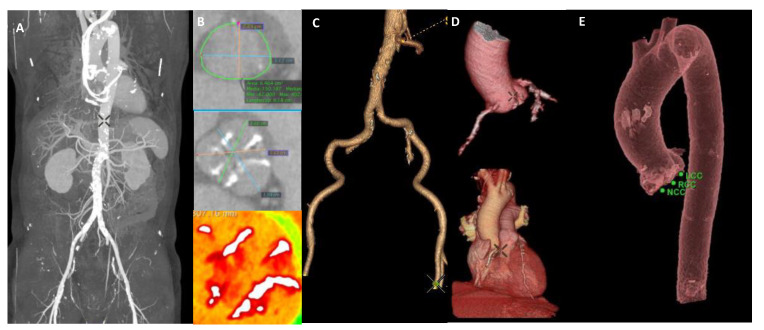
Pre-TAVI work-up MSCT analysis. (**A**) Transfemoral route: from skin to valve MSCT analysis. (**B**) Aortic valve MSCT analysis. (**C**) 3D reconstruction of femoral vascular accesses. (**D**,**E**) 3D reconstruction of aortic root, aortic arch, and descending aorta.

Multi-slice computed tomography (MSCT) plays a crucial role in the pre-procedural TAVI work-up by implementing specific acquisition protocols addressing different anatomical and procedural needs (Figure 1). MSCT imaging should be performed with intravenous iodine contrast injection to provide adequate and detailed anatomical information, especially for the aortic root and the iliofemoral arteries.

Preprocedural MSCT imaging should cover a large volume imaging dataset, extending from the proximal supra-aortic vessels to the area located below the femoral heads including the common femoral arteries where both the operative and non-operative access sites will be located.

To provide motion-free images of the aortic root and obtain the desired accuracy, MSCT imaging acquisition must be synchronized with the electrocardiogram (ECG) either by retrospective ECG gating or through the use of prospective ECG triggering [48].

However, it is not mandatory to acquire the entire aorta and iliofemoral arteries with ECG synchronization. Indeed, ECG-gated dataset acquisition is followed by a subsequent non-ECG-synchronized MSCT angiographic scan of the chest, abdomen, and pelvis to assess access route, which is mandatory in the TAVI work-up and planning. For this purpose, non-gated acquisitions may be preferable due to lower radiation exposure and because of faster volume coverage, which requires lower volumes of iodinated contrast medium [48].

MSCT-based TAVI planning relies on post-acquisition analysis: it may use multiplanar reconstruction, curve multiplanar reformats, and 3-dimensional (3D) volume-rendered images. The post-acquisition reconstructions are needed since the cardiac contractions make the aortic root and annulus dimensions change throughout the cardiac cycle [49]. The pulsatile changes lead to larger annular sizes in systole [50]. Moreover, prosthesis sizing algorithms use systolic measurements to avoid valve undersizing with measurements during diastole.

Acquired images of the cardiac cycle are analyzed using dedicated software. 3Mensio Medical Imaging (Pie Medica Imaging, Maastricht, the Netherlands), OsiriX DICOM Viewer (Pixmeo SARL, Bernex, Switzerland), and Horos (Horos Project, Geneva, Switzerland) are the most commonly used platforms.

The aortic valve annulus is not a discrete, separate anatomic structure. Much rather, the aortic annulus corresponds to a virtual plane formed by joining the three lowest points of the aortic valve leaflets (hinge point) within the left ventricle, aligned with the nadirs of the basal attachments of the native leaflets. It represents the inlet from the left ventricular outflow tract into the aortic root [51].

Long- and short-diameters, the planimetry of the area, and the circumference of the aortic annulus are then measured, together with the corresponding derived diameters.

The presence of valvular calcification and its localization requires a detailed description. Leaflet calcifications may be important to ensure prosthesis anchorage; conversely, excessive calcification in the prosthesis landing zone may favor the occurrence of paravalvular aortic regurgitation after implantation [52]. Finally, protruding sub-annular calcifications are relevant anatomical features due to their association with a higher risk of annular rupture [53].

In addition to annular sizing, MSCT analysis identifies the aortic root orientation and the appropriate orthogonal view onto the aortic annular plane to ensure a coaxial deployment [53]. The fluoroscopic angle of implantation predicted from preprocedural MSCT correlates well with 3D rotational angiography [53], reducing procedure time, contrast media volume, and radiation exposure throughout the stepwise optimization of the fluoroscopy unit. Various methods have been suggested for the determination of the optimal projection for TAV [53]. Balloon-expandable transcatheter heart valves are commonly centered and deployed in a perpendicular view of the aortic valve annulus, with a precise alignment of the coronary cusp [53]. Self-expanding THVs, instead, engage the aortic valve from the outer aortic curvature, and prior to deployment, foreshortening of the delivery catheter is usually eliminated by C-arm adjustments, frequently imposing a simultaneous deviation from the annular plane. In this setting, a recent paper by Ben-Shoshan et al. demonstrated that accurate planning during the pre-TAVI work-up through MSCT-based strategies such as “double-s” and ”cusps overlap” led to a high rate of procedural success (98%) with a very low rate of procedural complications including major bleedings, major vascular complications, and advanced kidney injury [53].

Moreover, Grodecki et al. [54] recently reported the correlation between quantitative CT angiography assessment of aortic valve tissue volume and composition. Indeed, it is possible to identify different valve tissue components using HU thresholds for calcific and non-calcific tissues. High-gradient aortic stenotic valves were characterized by increased calcific components, in terms of both absolute and relative volume. Conversely, low-flow, low-gradient AS patients presented a higher proportion of non-calcific tissue. The different aortic valve tissue compositions could explain the lesser flow resistance generated by fibrous and fibro-fatty tissue, which leads to lower trans-valve gradients. Additionally, the authors described a correlation between both total aortic tissue volume and the volume of non-calcific aortic valve tissue, but not calcific tissue alone, and 30-day MACEs after TAVI [54].

Further analysis provided a correlation between aortic valve calcific and non-calcific volumes with aortic valve severity [55]. Adding the quantification of both aortic valve fibrosis and calcification to the CT analysis may help to prevent aortic stenosis severity underestimation, particularly in females. Indeed, females present a higher predominance of non-calcific (fibrotic) valvular volume, with a higher fibro-calcific ratio [55]. Consequently, fibrocalcific volume, which correlated well with echocardiographic measurements, could be helpful to evaluate aortic stenosis severity [55].

Additionally, combined positron-emission-tomography (PET)/CT has emerged as a promising technique that might improve our pathophysiological understanding of AS. In fact, ^18^F-FDG (fluorodeoxyglucose) uptake is increased in aortic stenosis [56] and it is considered as a marker of inflammation and valvular calcification activity. Moreover, in a small sample trial, ^18^F-FDG uptake predicted disease progression and adverse clinical outcome [57].

A MSCT scan is essential to provide an adequate assessment of the access route. A detailed ‘skin-to-valve’ evaluation is crucial to understand the anatomy and plan the procedure. While the iliofemoral route is the most common access route for TAVI, the MSCT dataset should include the acquisition of the subclavian arteries and the mid-femoral arteries and the carotids and the circle of Willis, investigating possible alternative access routes.

Computed tomography provides thin-section isotropic volume data with a high spatial resolution that permits the identification of patients with an unsuitable iliofemoral vasculature for TAVI. Sheath-to-vessel ratio, defined as the ratio between the sheath outer diameter and the vessel minimal lumen diameter, is another parameter to consider in assessing the feasibility of a true percutaneous femoral approach, the need of a surgical cut-down, or predilatation of the iliac arteries [58].

The MSCT scan also identifies the presence of anatomical features related to a higher risk for vascular complications such as moderate or severe calcification, peripheral vascular disease, and vessel tortuosity [59].

The identification of concomitant high-risk features for vascular complications such as poor minimal lumen diameter, high burden of calcifications, and excessive tortuosity of the iliofemoral axis should prompt the operator to investigate for alternative access routes [60].

The trans-subclavian/trans-axillary route is the most common and preferred alternative access route in patients presenting with challenging or prohibitive transfemoral access [61]. Meticulous attention should be given to the subclavian ostia, which can have calcifications that increase the risk of cerebral embolism. The aorto-ventricular angle is a measurement used to quantify the curvature of the proximal aorta, aortic valve, and left ventricular outflow tract. The optimal aorto-ventricular angle is less than 70° for a left subclavian approach and less than 30° for a right subclavian approach.

The trans-caval technique is a novel favorable access strategy access route in patients presenting highly calcified iliofemoral vessels with an inadequate minimal lumen diameter. The MSCT should evaluate the anatomical relationship between the inferior vena cava and the abdominal aorta at the level of L3–L4. In particular, the close proximity between the two vessels should be free from calcifications [62].

The trans-carotid/transapical/transaortic are favorable alternative access routes due to their close proximity to the aortic annulus. In the transaortic approach, attention should be paid to the height of the puncture in relation to the aortic annulus. However, the presence of aortic calcifications, increasing the risk of cerebral embolization, should be considered a contraindication. Of note, MSCT can provide detailed information about the length of the ascending aorta as well as evaluate the distribution and burden of calcifications. In the transapical approach, the MSCT is useful to identify the level of the access (usually between the fifth and the sixth intercostal space) to rule out an apical thrombus, and to evaluate additional parameters such as the distance between the virtual basal ring and the apex, thickness of the apical wall. Finally, the trans-carotid access ensures co-axiality, especially when the right carotid artery is used [63]. Of note, in this particular setting, MSCT provides key information regarding the diameter of the carotid arteries as well as the presence and extent of any atherosclerotic disease, which may impair cerebral blood flow due to the use of large-bore catheters.

Among the TAVI population, chronic kidney disease is a common presentation, with up to more than two-thirds of the patients having an estimated glomerular filtration rate (eGFR) below 60 mL/min/1.73 m^2^ [64]. Consequently, the prevention of further renal deterioration should be one of the priorities during work-up, minimizing the need for contrast media. Nevertheless, specific preventive strategies such as periprocedural hydration are required for those with eGFR <30 mL/min/1.73 m^2^ [65].

Considering the wide range of MSCT acquisition and the elderly population, about 20% of preprocedural TAVI work-up MSCT has potentially malignant findings [66,67], which may alter the aortic valve treatment course. These incidental findings are associated with poorer survival in those without incidental findings; however, if these findings are addressed effectively in the best ongoing care, the differences in survival between those with and without incidental findings become negligible [67].

## 4. Management of Coronary Artery Disease in Severe Aortic Stenosis

### 4.1. Assessment of Coronary Artery Disease

Coronary artery disease (CAD) and degenerative calcific aortic stenosis, particularly in elderly-high risk patients, are frequently associated [68] with a prevalence of about 50% [69]. The prevalence of CAD showed a progressive reduction in parallel with the decrease in mean age and surgical risk in randomized controlled trials [4,5,9].

Of note, about 50% of TAVI candidates with CAD exhibit multivessel disease [70], with the involvement of the left main and left anterior descending artery in 11% and 50% of patients, respectively [71].

About 12% of TAVI recipients [5] included in randomized controlled trials underwent PCI before TAVI, and this proportion reached 25% in real-world TAVI registries [5].

The impact of CAD in patients undergoing TAVI, its correct management, the clinical relevance, and its prognostic role is still a matter of debate [72] (Table 4).

To date, most likely due to the different demographics and comorbidities, randomized trials have failed to demonstrate a clear beneficial effect of percutaneous coronary intervention (PCI) in stable CAD patients [73], while stronger data are available showing the benefits on long-term clinical outcomes following surgical coronary revascularization in the context of SAVR population [74].

Current guidelines state that in the presence of severe left main (LM) or proximal coronary artery disease in patients with or without angina, percutaneous revascularization before TAVI is reasonable [8].

In this regard, two recent meta-analyses showed contradictory results regarding the association between CAD and clinical outcomes post-TAVI [71,75].

Coronary angiography still represents the standard examination to assess CAD in the pre-TAVI work-up.

Coronary angiogram is recommended before valve surgery in patients with severe aortic stenosis and history of CAD, suspected myocardial ischemia, left ventricular dysfunction, one or more cardiovascular risk factors, or in men older than 40-year-old or postmenopausal women [6].

A recent study proposed the use of noninvasive coronary imaging such as computed tomography coronary angiography (CTCA) as guidance to decide whether to perform coronary angiography (CA) [76]. Based on this strategy, CA was performed in only 24% of the TAVI candidates without a clinical negative impact in those cases where CA was avoided. Further studies showed that implementing CTCA in the TAVI work-up could decrease the number of coronary angiographies by 37% [77]. However, heavy calcifications generally resulted in an increase in false-positive results, therefore representing a limit of the technique in younger patients with a much lower probability of CAD and a lower degree of coronary artery calcification [78].

Previous studies have compared the performance of coronary CTCA with CA for the detection of significant coronary stenosis in the context of pre-TAVI work-up, showing an excellent performance in terms of negative predictive value, although associated with poor specificity [79,80]. Similar sensitivity (95% vs. 99%) but lower specificity (65% vs. 88%) and a higher contrast volume have been associated with CTCA during the pre-TAVI work-up, compared to subjects without aortic stenosis [77,81].

Indeed, randomized trials failed to demonstrate a clear beneficial effect of PCI in stable CAD patients [73], and current guidelines state that performing PCI in the presence of coronary artery stenosis >70% in proximal coronary segments during the pre-TAVI work-up lacks scientific evidence [6]. As a result, the best management of CAD in TAVI candidates is unclear [82].

In the presence of coronary lesion without evidence of ischemia in the corresponding myocardial territory, current guidelines state that revascularization should be guided by the hemodynamic functional assessment [83].

The functional assessment of coronary obstructions by means of FFR has been demonstrated to be feasible and reliable in patients with severe aortic stenosis [84,85] (Table 5).

The reliability and the safety of FFR measures have been questioned because of the impaired capacity to achieve maximal hyperemia and because of the potential risk of adenosine administration in this subset of patients. However, no complications related to the administration of intracoronary adenosine or the use of the pressure wires have been reported [84].

It has been demonstrated that aortic valve stenosis influences functional coronary artery indexes [90]. Moreover, the immediate improvement in the coronary physiological reserve induced by the aortic valve replacement has been demonstrated [91].

Indeed, FFR values measured after valve replacement may be more accurate to evaluate the need for myocardial revascularization compared with values obtained during preintervention diagnostic examinations, particularly among angiographically intermediate stenosis [84].

Despite the demonstration of clinical equivalence of FFR and iFR in patients with CAD and without aortic valve disease, this is not true for patients with severe aortic stenosis candidates to TAVI.

A “hybrid iFR-FFR strategy” was proposed to spare the majority of patients from adenosine [88]. However, a lower iFR threshold for predicting FFR ≤0.8 is suggested in aortic stenosis patients. Indeed, a 0.83 iFR cut-off for matching the 0.8 FFR values has been reported in the “hybrid iFR-FFR” aortic stenosis patients tailored strategy [88].

#### 4.1.1. PCI before TAVI

To date, there are still inconsistent findings on the prognostic significance of CAD and the effect of revascularization before TAVI, even when such strategy is driven by the use of the Syntax Score [72].

Limited data are available on the safety of PCI in the presence of untreated severe aortic stenosis. In this setting, intraprocedural complications may have a relevant hemodynamic impact [71].

PCI before TAVI should be considered in patients presenting with an acute coronary syndrome, in patients with left main stem, or critical ostial lesions. Moreover, anatomical considerations such as unfavorable aortic annulus anatomy or valve-in-valve procedures should be taken into account.

A potential disadvantage of PCI upstream to TAVI is the need for dual antiplatelet therapy, which could increase the hemorrhagic risk [92].

Additionally, recent data demonstrated a significantly higher risk of contrast-induced acute kidney injury (CI-AKI) in patients who underwent TAVI and coronary procedures in a staged strategy. Conversely, a concomitant strategy (i.e., TAVI and PCI performed in the same procedure) demonstrated a lower risk of CI-AKI and the difference between these two strategies was mainly driven by the incidence of either pre-TAVI coronary angiography or ad-hoc PCI [93].

Until the results of ongoing trials are available, patients with CAD undergoing TAVI will require a comprehensive assessment by a multidisciplinary Heart Team, ensuring individualized management based on their clinical and angiographic findings.

#### 4.1.2. PCI after TAVI and Coronary Re-Access 

As previously mentioned, the incidence of CAD in patients undergoing TAVI is high, even in those who are at intermediate risk [9,94]. Given the progressive nature of CAD, a significant proportion of these patients will require coronary angiography and possibly PCI in the years following the aortic valve implantation. 

Performing TAVI before PCI could be advantageous as it improves left ventricle hemodynamics [95] and decreases the risk of intraprocedural hemodynamic instability related to aortic stenosis, particularly in complex PCI scenarios. However, the presence of the prosthesis may imply some technical challenges in re-accessing the coronary ostia, as demonstrated by Barbanti et al. [96] in the REACCESS study. In this single-center, prospective registry, the authors found that a combination of (1) Evolut TAV, (2) a higher TAV–sinus of Valsalva relation, and (3) implantation depth predicted the risk for unsuccessful coronary cannulation after TAVI with high accuracy (area under the curve: 0.94; *p* < 0.01).

Recent data [97,98] may suggest design matters in terms of ease of coronary ostia re-access, with the self-expanding valve associated with greater challenges in coronary angiography and PCI post-TAVI. 

In addition to anatomical factors, valvular prosthesis-specific factors could impact coronary re-access post-TAVI.

Indeed, device positioning and orientation during valve deployment have recently achieved attention. In particular, commissural alignment of THV in relation to coronary artery ostia seems to play a relevant role in the ease of coronary re-access.

The possibility to control and orient the prosthesis valve commissural alignment, especially in lower-risk and younger patients, is crucial to avoid overlap between coronary ostia and THV commissures [99].

The objective is to optimize the neo-commissural alignment to reduce the risk of overlap with coronary artery ostia and to facilitate the coronary re-access [72]. Coronary re-access after TAVI should begin with an ascending aortogram in order to detect the coronary artery ostia. The choice of catheter and technique to attempt coronary cannulation is affected by the geometry, features, dimension, and level of implantation across the aortic annulus of THV.

Yudi et al. [72]. proponed an algorithm for diagnostic catheter selection and guided catheter selection, in order to facilitate coronary re-access after TAVI.

In challenging cases, advancing a coronary wire through the THV cell and using it as a rail may be helpful in obtaining stable coronary cannulation. Moreover, using a smaller catheter, a telescopic catheter system or a guide extender could allow for easier coronary re-access and avoid having difficulty removing the guide [100]. Given the possibility of coronary re-access in patients with established or intermediate CAD lesions, especially in younger and lower-risk patients, a Heart Team discussion on the management of CAD in patients with severe AS is key, and it warrants a tailored approach focusing on (1) the consistent treatment of both diseases (percutaneous versus surgical); (2) timing of coronary intervention; (3) careful valve selection (which may affect coronary re-access), and (4) Heart Team directed revascularization.

### 4.2. Coronary Artery Occlusion

#### 4.2.1. Native Aortic Valve

Coronary artery occlusion (CAO) following the displacement of the calcified native valve leaflets over the coronary ostia represents a potential complication of transcatheter aortic valve implantation (TAVI), with a reported incidence usually <1% (ranging from 0% to 4.1%) [101,102].

In native aortic valves, coronary ostia obstruction is related to two main procedural factors: (1) The main mechanism is the displacement of a bulky calcified native valve over a coronary ostium; and (2) as a consequence of the possible obstruction by a portion of the THV frame/sealing cuff located directly over a coronary ostium.

Some anatomic features (narrow sinus of Valsalva, bulky leaflet calcifications, low-lying coronary ostia) have been recognized as factors involved in its pathogenesis. 

The use of multimodality imaging should play a central role in the pre-TAVI word-up, particularly in highlighting a high-risk feature such as the bulkiness of the native cusps, the height of the coronary ostia, and the dimensions of the sinus of Valsalva. 

In routine practice, the analysis of multidetector computed tomography (MDCT)-derived imaging, through the 3D reconstruction of the aortic root, provides an extremely reliable tool for assessing dimensions and calcium distribution [103].

#### 4.2.2. Valve-in-Valve

TAVI indication is progressively moving toward younger patients presenting with lower surgical risk and longer life expectancy [97]. Repeat TAVI is an attractive solution for failing transcatheter heart valves. In such a scenario, the newly implanted THV effectively creates a tube graft/cylindrical cage within the ascending aorta by pinning open the leaflets of the first THV, leading to the possible sequestration of the sinuses of Valsalva, which may impair both coronary cannulation and coronary flow. A MSCT-based analysis (*n* = 137) by Rogers et al. [104] showed that this phenomenon was mainly driven by (1) THV extending above the sinotubular junction, and (2) a THV commissural suture post located directly in front of the coronary ostium.

In self-expanding THVs, even if coronary perfusion is maintained around the nested THVs, the extension of the tube graft above the sinotubular junction (STJ) may prohibit future coronary access, mainly due to the supra-annular leaflet position and tall stent frame characterizing these prostheses.

Coronary obstruction is three- to four-fold more common after TAVI in degenerated surgical bioprostheses than native valve TAVI.15. Recently, Dvir and colleagues reported a coronary obstruction incidence of 3.5% of patients [105]. The main predisposing factor in the setting of aortic valve-in-valve procedures is the proximity of the coronary ostia to the anticipated final position of the displaced bioprosthetic leaflets after THV implantation. Therefore, predisposing factors for coronary obstruction may include a supra-annular bioprosthetic valve, a narrow and low-lying sinotubular junction, bulky bioprosthetic leaflets, low-lying coronaries in narrow aortic roots, and reimplanted coronaries [105]. In particular, stentless bioprosthetic valves or those that are internally stented (e.g., Mitroflow, Sorin; Trifecta, St Jude Medical) may be at a higher risk because the leaflets of these bioprostheses may extend outward in a tubular fashion after valve implantation beyond the surgical device frame [105,106].

In this setting, a crucial aspect of work-up is the in-depth knowledge of surgical prosthesis previously implanted

Therefore, a meticulous understanding of the differences in surgical heart valve design is of paramount importance to allow for the optimal selection of THV and the prevention of CAO.

Conversely, the choice of the prosthesis type for valve-in-valve TAVI procedures should be individualized for each patient. Indeed, the assessment of the risk for CAO may indeed influence THV selection. THV devices that could be immediately retrieved after partial device implantation may be the strategy of choice (e.g., Lotus, Portico, Evolut-R, etc.) to prevent such complications [107].

The “chimney/snorkeling” bail-out technique could be used in the case of sudden CAO in patients considered at high risk and who underwent coronary protection during TAVI [108].

A new technique called BASILICA (bioprosthetic or native aortic scallop intentional laceration to prevent iatrogenic coronary artery obstruction) was developed and first performed in 2017 to prevent CAO, especially in the case of valve-in-valve TAVI in degenerated surgical bioprostheses.

The BASILICA technique is based on an intentional valve leaflet laceration in front of the coronary ostium, addressing the pathophysiology of CAO due to leaflet’s displacement. After TAVI, the sliced leaflet will be split, creating a triangular space that allows for preserved blood flow through the coronary ostium to the coronary artery [109]. 

However, it is crucial to consider and assess the risk of delayed CAO, which could occur after removing protective wires from coronary arteries [110].

Precisely, an IVUS-based strategy to evaluate the relationship between coronary ostium degenerated valvular leaflets, and THV frame in the para-ostial space has been proposed [111]. Indeed, the IVUS analysis may help in detecting the degenerated leaflet footprint on the coronary ostium in the case of partial obstruction and guide the operator in the choice of protection strategy.

## 5. Role of the Heart Team 

**Figure 2:**
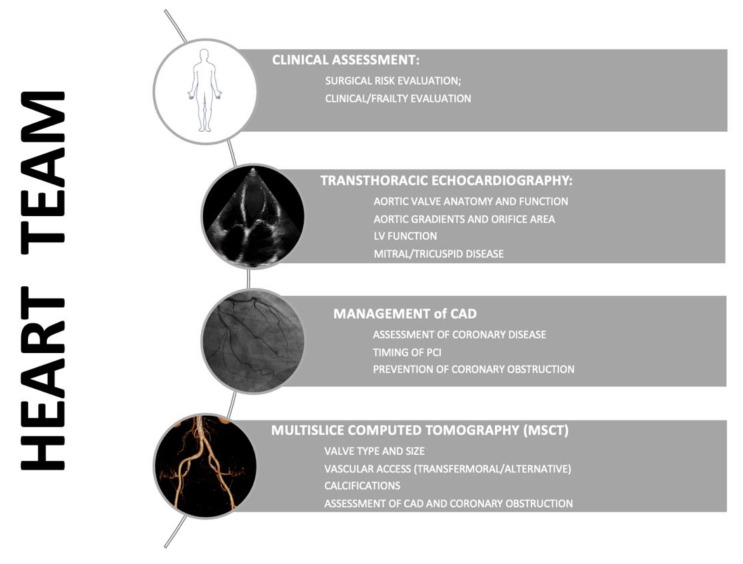
Pre-TAVI work-up and Heart Team.

The concept of the Heart Team as a collaborative way of integrating the expertise of both interventional cardiologists and cardiothoracic surgeons to deliver the highest, most appropriate care in a timely fashion while minimizing procedural risks was developed in the early 2000s for patients presenting with highly complex cardiac disease. In particular, to address the deficiencies of risk models previously described to guide the management of aortic valve disease, in recent years, both European and American medical and scientific organizations have strongly endorsed the role of the Heart Team in making the treatment decision [112], and providing patients with a better quality of care. For such a reason, the guidelines stress that aortic valve interventions should be offered only by centers with both cardiology and cardiac surgery departments on-site that are capable of supporting a structured collaboration (Figure 2).

A critical role in putting the Heart Team at the core of clinical decision was played by the ESC Working Group on Valvular Heart Disease, which recommended the creation of Heart Valve Clinics (HVC) to concentrate the expertise in heart valve disease (HVD) to provide highly standardized evaluation, care, and education to such challenging patients. Thus, patients referred to the Heart Team should be evaluated by an interventional cardiologist and cardiac surgeon specialized in valve disorders in order to provide a tailored approach, based upon a careful individual evaluation of technical suitability, together with the risks and benefits of each modality. Besides the two key figures described above, a comprehensive assessment requires the creation of a multidisciplinary team integrating the expertise of other physicians such as:

- clinical cardiologist;
- anesthesiologist;
- imaging specialists (echocardiographer, radiologist);
- geriatrician; and
- cardiac rehabilitation specialist.

The multidisciplinary team should be characterized by fruitful interaction among specialists, particularly for pre-procedure careful individual evaluation and patient selection to avoid futility [113].

The decision-making process should explicitly take account of the anatomical severity of aortic stenosis and that this severity accounts for the patient’s symptoms, the patient’s expected general prognosis and that related to the aortic valve disease, the risks and late consequences of valvular intervention, the patient’s wishes and quality of life as well as the local resources available and the local procedural outcomes. The Heart Team should discuss the treatment options with the patient, who can then make an informed choice [7].

The work of the Heart Team should lead to the following goals [112]: (1) confirmation of the correctness of the diagnosis and the indication for the intervention; (2) the elaboration of a treatment proposal; and (3) the production of written documentation of the motivation for the treatment proposal in the case of medical, catheter-based, or surgical therapy.

## 6. Conclusions

The management of AS patients should always have the appropriateness of care as its final goal. The highly demanding care and the complexity of the disease in terms of diagnosis, treatment decision, procedural execution, and follow-up evaluation require the integrated collaboration of valvular heart disease experts working in dedicated centralized institutions. Such an environment is an essential condition to perform a thorough preprocedural work-up as mandatory to align clinical indications and expectations, dedicated physicians, and patients in preparation for the intervention. Once again, a carefully planned strategy should be regarded as the key step leading to a successful procedure, consistent results, and the satisfaction of patients’ needs.

## 7. Future Perspectives 

The TAVI environment has been changing at a fast pace over the last two decades. Future progressive expansion in indications may be expected as percutaneous treatment becomes less and less invasive while maintaining and even improving the current outcome measures of safety and efficacy. In light of such continuous and rapid reshaping of the technological and clinical landscape, the role of the work-up and the Heart Team will be even more crucial to guarantee the appropriateness of care, treatment decision, and procedural delivery.

## Figures and Tables

**Table 1:** Procedure-specific risk factors for interventions not incorporated into the existing risk scores.

	TAVI	SAVR
Technical or anatomic	Aorto-iliac occlusive disease precluding transfemoral approach	Prior mediastinal radiation
	Aortic arch atherosclerosis	Ascending aortic calcification (porcelain aorta)
	Extensive LV outflow tract calcification	
	Low-lying coronary arteries	
	Severe MR or TR	
	Basal septal hypertrophy	
Comorbidities	Severe COPD or home oxygen therapy	
	Pulmonary hypertension	
	Severe RV dysfunction	
	Hepatic dysfunction	
	Frailty	

LV: left ventricle; RV: right ventricle; MR: mitral regurgitation; TR: tricuspid regurgitation; COPD: chronic obstructive pulmonary disease.

**Table 2:** Summary of frailty scores used in clinical practice during pre-TAVI work-up.

Score	Functional Status	Mental	Blood Tests	Nutritional	Therapy	Social Support
**CGA** **( Comprehensive Geriatric Assessment)**	Activity of daily living (BADLs, IADLs, AADLs) Mobility Gait speed Balance	Cognitive impairment Mood disorders		Weight Appetite	Polypharmacy	Social and financial support
**EFT** **(Essential FrailtyToolset)**	Chair rises	Cognitive impairment	Albumin Hemoglobin			
**Frailty Index**	Strength Mobility Activity of daily living Comorbidities	Cognitive and mood impairment		BMI Weight loss	Help taking medications	Help with finances
**Fried**	Weight loss Weakness (handgrip strength) Exhaustion Physical activity Slowness (transit time)					
**SPPB** **(Short physical performance battery)**	Side by side stand Semi-tandem stand Tandem stand					

BMI: body mass index; BADLs: basic activities of daily living scale; IADLs: instrumental activities of daily living scale; AADLs: advanced activities of daily living scale.

**Table 3:** Pre-TAVI work-up: transthoracic echocardiography assessment.

	Valve Anatomy	Valve Hemodynamics
**Mild aortic stenosis**	Mild to moderate leaflet calcification or fibrosis of a bicuspid or trileaflet valve with some reduction in systolic motion	Aortic Vmax 2.0–2.9 m/s or mean ΔP < 20 mmHg
**Moderate aortic stenosis**		Aortic Vmax 3.0–3.9 m/s or mean ΔP 20–39 mmHg
**Severe aortic stenosis**	Severe leaflet calcification or fibrosis with severely reduced leaflet opening	Aortic Vmax > 4 m/s or ΔP > 40 mmHg or AVA typically < 1.0 cm^2^
***High-gradient***		Aortic Vmax > 4 m/s and AVA < 1.0 cm^2^
***Low-flow, low-gradient***		Reduced ejection fraction LVEF < 50% AVA < 1.0 cm^2^ Resting aortic ΔP < 40 mmHg or aortic Vmax < 4 m/s Stroke volume index (SVi) < 35 mL/m^2^ Dobutamine stress echocardiography shows AVA < 1.0 cm^2^ with Vmax > 4 m/s at any flow rate Preserved ejection fraction (Paradoxycal low-flow severe AS) LVEF > 50% AVA < 1.0 cm^2^ ΔP < 40 mmHg or aortic Vmax < 4 m/s Stroke volume index (SVi) < 35 mL/m^2^
***Normal-flow, low gradient***		AVA < 1.0 cm^2^, ΔP < 40 mmHg, LVEF ≥ 50%, and SVi < 35 mL/m^2^

AVA: aortic valve area; LVEF: left ventricle ejection fraction; SVi: stroke volume index.

**Table 4:** Ongoing randomized controlled trials investigating CAD management in TAVI patients.

Study	NOTION-3 (NCT03058627)	TCW (NCT03424941)	FAITAVI (NCT03360591)	CT-CA (NCT03291925)	ACTIVATION (ISRCTN75836930)
Sample size (pts)	452	328	320	200	310
Population	Patients with severe aortic stenosis selected for TAVI and at least one coronary stenosis with FFR ≤0.80 or diameter stenosis >90% in a coronary artery ≥2.5 mm	Patients age <70 years with severe AS feasible for treatment by both TF or TSc approach TAVI as well as conventional SAVR, and ≥2 de novo coronary lesions ≥50% diameter stenosis on main artery or side branch >2 mm or single LAD lesion >20 mm length or involving a bifurcation, feasible for treatment with CABG as well as PCI	Patients with severe AS with the indication of TAVI and at least one coronary stenosis >50% at angiography	Patients with symptomatic severe AS accepted for TAVI	Patients with symptomatic severe AS accepted for TAVI, and ≥1 proximal stenosis of ≥70% in a major epicardial artery deemed suitable for PCI
Intervention/comparator	TAVI only vs. TAVI þ FFR-guided complete revascularization	FFR-guided PCI and TAVI vs. CABG and SAVR	Physiologically-guided strategy (PCI of lesions with FFR <0.80 vs. angiographically guided strategy (PCI of all lesions >50% by visual estimation of major branches >2.5 mm)	Selective invasive angiography Number of patients enrolled in the based on CT/coronary CTA study of all those that are eligible imaging vs. systematic invasive angiography	Pre-TAVI PCI vs. no pre-TAVI PCI
Outcomes	All-cause mortality, myocardial infarction, or urgent revascularization at 1 year	Composite of all-cause mortality, myocardial infarction, disabling stroke, unscheduled clinically- driven target vessel revascularization, valve reintervention, and life threatening or disabling bleeding at 1 year	Composite of all-cause death, myocardial infarction, stroke, major bleeding and target vessel revascularization at 1 year	Number of patients enrolled in study of all those that are eligible	Mortality and rehospitalization at 1 year

TAVI: transcatheter aortic valve implantation; FFR: fractional flow reserve; TF: transfemoral; TSc: transsubclavian; SAVR: surgical aortic valve replacement; AS: aortic stenosis; LAD: left anterior descending; CABG: coronary artery bypass graft; PCI: percutaneous coronary intervention; CT: computed tomography; CTA: computed tomography angiography.

**Table 5:** Studies evaluating the use of invasive physiology during the pre-TAVI work-up.

Study	Population	Intervention	Follow-up
Pesarini et al., 2016 [84]	133 lesions (*n* 1⁄4 54)	PCI performed in 19/133 lesions	At 30 days: No sustained angina or hypotension, myocardial infarction, or heart failure
Stanojevic et al., 2016 [86]	82 lesions (*n* 1⁄4 72)	PCI performed in 37/82 lesions	At a median follow up of 19 14 months after TAVI:4 ACS, no TVR or TLR
Ahmad et al., 2018 [87]	30 lesions (*n* = 28)	N/A	N/A
Scarsini et al., 2018 [88]	141 (lesions) (*n* = 62)	PCI performed in 19/141 lesions	At 30 days: No death or new coronary revascularization
Yamanaka et al., 2018 [89]	116 vessels (*n* 1⁄4 95)	N/A	N/A

TAVI: transcatheter aortic valve implantation; ACS: acute coronary syndrome; TVR: target vessel revascularization; TLR: target lesion revascularization; N/A: not applicable.
